# Dramatic Consequences of Reducing Erythrocyte Membrane Cholesterol on Plasmodium falciparum

**DOI:** 10.1128/spectrum.00158-22

**Published:** 2022-02-23

**Authors:** Avantika I. Ahiya, Suyash Bhatnagar, Joanne M. Morrisey, Josh R. Beck, Akhil B. Vaidya

**Affiliations:** a Center for Molecular Parasitology, Institute for Molecular Medicine and Infectious Disease, Department of Microbiology and Immunology, Drexel University College of Medicine, Philadelphia, Pennsylvania, USA; b Department of Biomedical Sciences, Iowa State University, Ames, Iowa, USA; University of Illinois at Urbana Champaign

**Keywords:** malaria, *Plasmodium falciparum*, parasite extrusion, cholesterol dynamics, PfATP4 and PfNCR1 inhibitors, vomocytosis, parasite expulsion

## Abstract

Cholesterol is the most abundant lipid in the erythrocyte. During its blood-stage development, the malaria parasite establishes an active cholesterol gradient across the various membrane systems within the infected erythrocyte. Interestingly, some antimalarial compounds have recently been shown to disrupt cholesterol homeostasis in the intraerythrocytic stages of Plasmodium falciparum. These studies point to the importance of cholesterol for parasite growth. Previously, reduction of cholesterol from the erythrocyte membrane by treatment with methyl-β-cyclodextrin (MβCD) was shown to inhibit parasite invasion and growth. In addition, MβCD treatment of trophozoite-stage P. falciparum was shown to result in parasite expulsion from the host cell. We have revisited these phenomena by using live video microscopy, ultrastructural analysis, and response to antimalarial compounds. By using time-lapse video microscopy of fluorescently tagged parasites, we show that MβCD treatment for just 30 min causes dramatic expulsion of the trophozoite-stage parasites. This forceful expulsion occurs within 10 s. Remarkably, the plasma membrane of the host cell from which the parasite has been expelled does not appear to be compromised. The parasitophorous vacuolar membrane (PVM) continued to surround the extruded parasite, but the PVM appeared damaged. Treatment with antimalarial compounds targeting PfATP4 or PfNCR1 prevented MβCD-mediated extrusion of the parasites, pointing to a potential role of cholesterol dynamics underlying the expulsion phenomena. We also confirmed the essential role of erythrocyte plasma membrane cholesterol for invasion and growth of P. falciparum. This defect can be partially complemented by cholesterol and desmosterol but not with epicholesterol, revealing stereospecificity underlying cholesterol function. Overall, our studies advance previous observations and reveal unusual cell biological features underlying cholesterol depletion of the infected erythrocyte plasma membrane.

**IMPORTANCE** Malaria remains a major challenge in much of the world. Symptoms of malaria are caused by the growth of parasites belonging to *Plasmodium* spp. inside the red blood cells (RBCs), leading to their destruction. The parasite depends upon its host for much of its nutritional needs. Cholesterol is a major lipid in the RBC plasma membrane, which is the only source of this lipid for malaria parasites. We have previously shown that certain new antimalarial compounds disrupt cholesterol homeostasis in P. falciparum. Here, we use live time-lapse video microscopy to show dramatic expulsion of the parasite from the host RBC when the cholesterol content of the RBC is reduced. Remarkably, this expulsion is inhibited by the antimalarials that disrupt lipid homeostasis. We also show stereospecificity of cholesterol in supporting parasite growth inside RBC. Overall, these results point to a critical role of cholesterol in the physiology of malaria parasites.

## INTRODUCTION

A salient feature of malaria parasites is their dependence on the host to fulfill their nutrient requirements. In addition to various nutrients, *Plasmodium* salvages lipids and fatty acids from the host. While the parasite possesses pathways for synthesizing and modifying lipids (1, 2), it lacks the machinery for *de novo* cholesterol synthesis (3–6). Unlike the intrahepatic stages where the parasites have access to copious amounts of cholesterol (7), blood-stage parasites can only access cholesterol present in the erythrocyte membrane (8). Recent studies from our laboratory have identified antimalarials that inhibit two parasite plasma membrane (PPM) transporters, PfATP4 and PfNCR1, which disrupt cholesterol and lipid homeostasis in the PPM (9, 10). Upon treatment with these compounds, there is a rapid accumulation of cholesterol in the PPM, rendering the parasites sensitive to the cholesterol-dependent detergent saponin. This was observed as the loss of cytosolic proteins in drug-treated parasites exposed to saponin. This effect was reversible, indicating an active mechanism of cholesterol dynamics within the parasite (9). These unexpected consequences of exposure to novel antimalarials suggest the presence of mechanisms that influence cholesterol transport within intraerythrocytic P. falciparum. Previously, it was shown that intraerythrocytic P. falciparum growth requires a supply of fatty acids that cannot be substituted with lipids and cholesterol from serum-derived lipoproteins (11). Furthermore, studies from the Haldar laboratory have shown that cholesterol in the erythrocyte plasma membrane is essential for invasion and growth of P. falciparum. Interestingly, reduction of cholesterol from the erythrocyte plasma membrane by treatment with methyl-β-cyclodextrin (MβCD) resulted in extrusion of the trophozoite-stage parasites (12).

Given the observations regarding the effects of new antimalarials on cholesterol dynamics and the consequences of cholesterol reduction from the infected erythrocyte plasma membrane, we wished to examine a potential link between these phenomena. We have used live fluorescence video microscopy, transmission electron microscopy, and treatment with antimalarials to revisit previous findings regarding the consequences of MβCD treatment of intraerythrocytic P. falciparum. Results from our experiments confirm and extend these observations and raise important mechanistic questions regarding the role of cholesterol in P. falciparum biology.

## RESULTS

### Dramatic expulsion of trophozoite-stage parasites upon treatment with MβCD.

**FIG 1:**
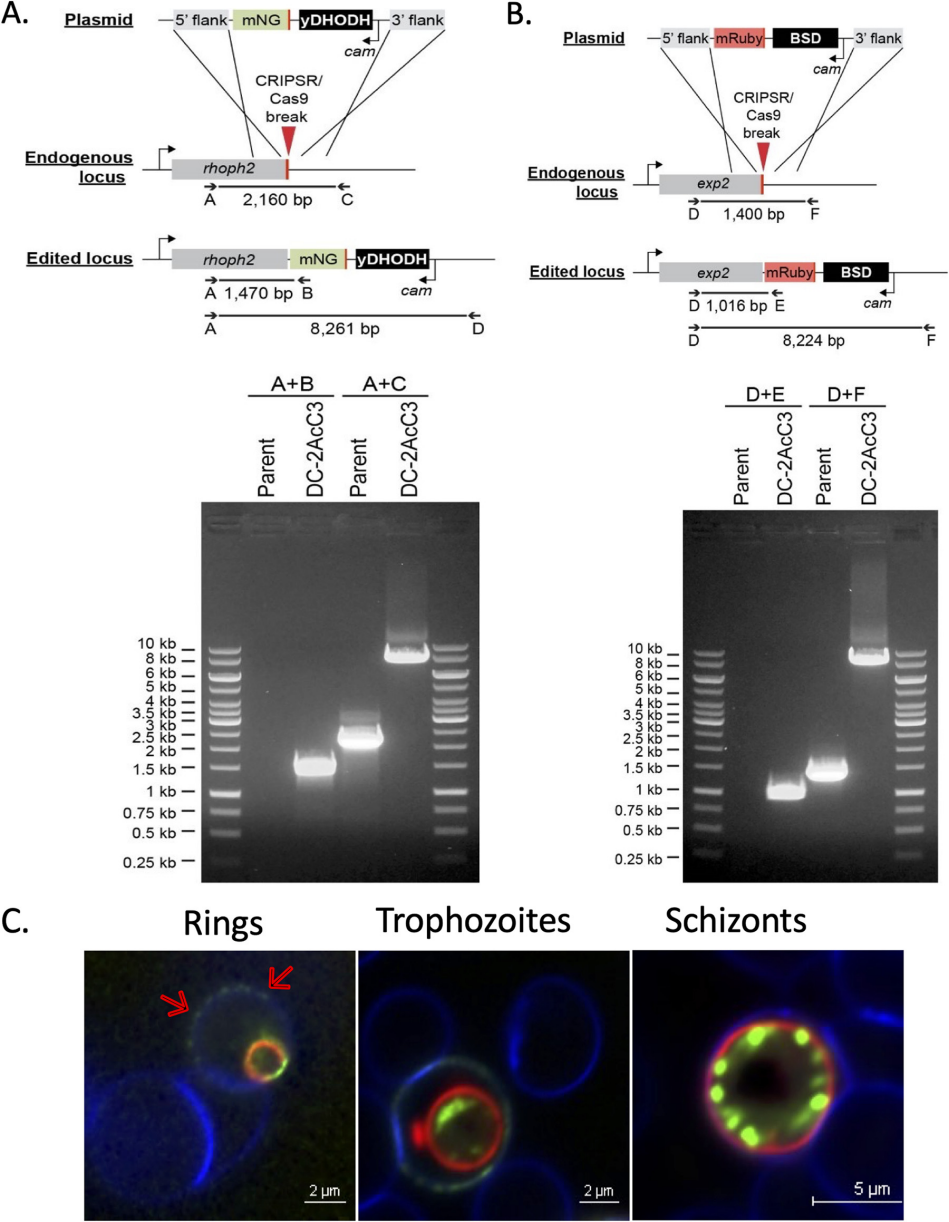
Generation of the NF54 Rhoph2/EXP2 line. (A) Strategy for tagging *rhoph2* with mNeonGreen (mNG) and gel image indicating presence of clonal population expressing *rhoph2-mNG*. (B) Strategy for the tagging *exp2* with *mRuby* tag and agarose gel image indicating the presence of clonal population expressing *exp2-mRuby*. Sequences of the diagnostic primers A, B, C, and D are provided in Materials and Methods. (C) Live fluorescence microscopy images indicating the expected localization of the tagged proteins. Red arrows indicate the dual localization of RhopH2-mNG on the RBC membrane as well as the parasite in the rings and trophozoites, while in the schizonts, the protein is localized to rhoptries. The EXP2-mRuby marks the PVM of the parasite. Erythrocyte membrane is stained with WGA-Alexa 350 (blue).

**FIG 2:**
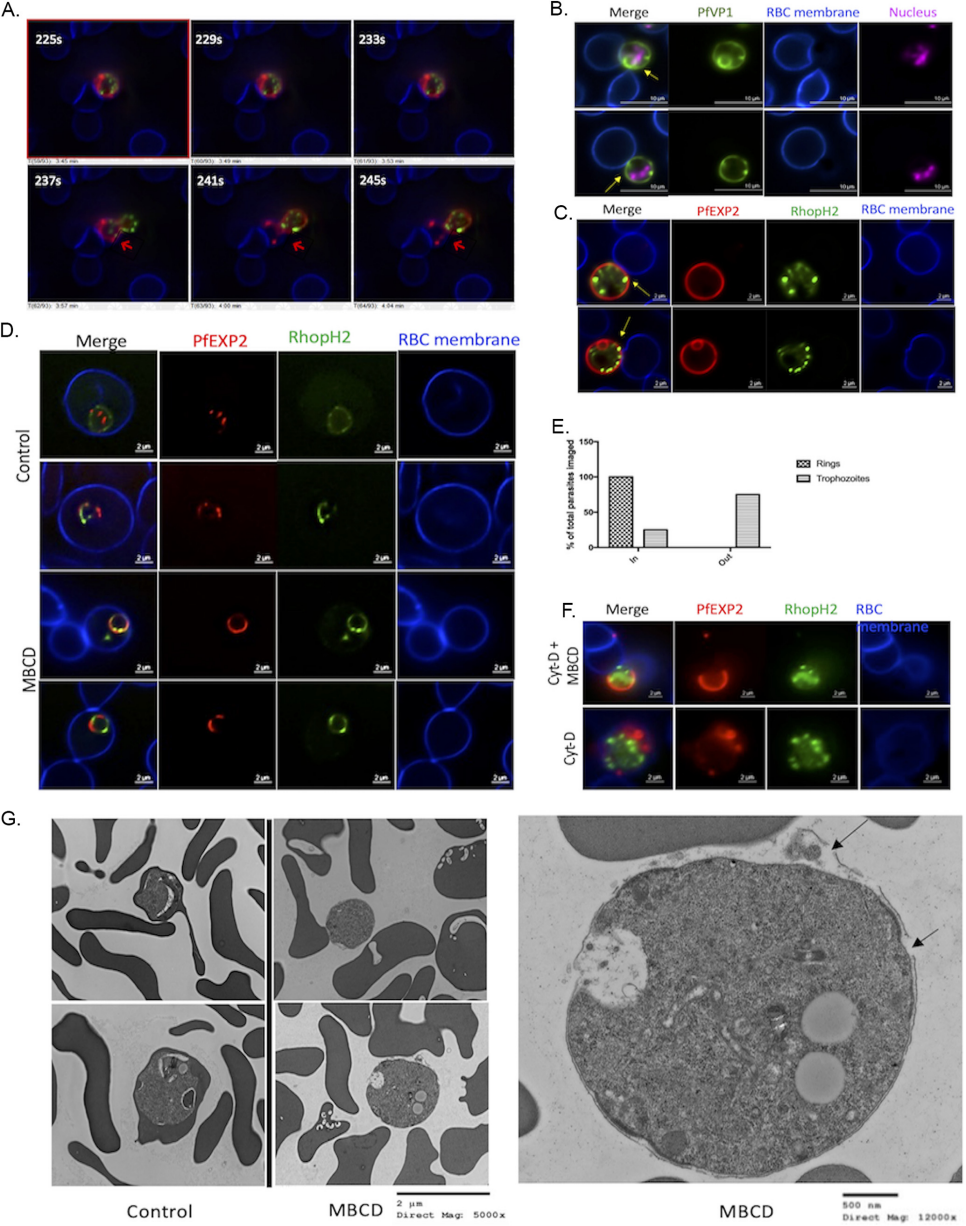
Trophozoites extrude out from erythrocyte upon treatment with MβCD. (A) Still frames from Video S1 in the supplemental material from time-lapse video microscopy. Parasites extrude out from the erythrocyte with the PVM still tethered to the erythrocyte membrane without causing the lysis of erythrocytes (indicated by arrows). (B) Representative images (>50) of the PfVP1-mNG line after MβCD treatment. Nucleus is stained with SYTO deep red (pink). (C) Representative images (>50) of the NF54 RhopH2/Exp2 line after MβCD treatment. Erythrocyte membrane is stained with WGA-Alexa 350 (blue). Infected erythrocytes were subjected to live fluorescence microscopy after treatment with MβCD. Parasites extrude out from the erythrocyte without causing lysis of the erythrocyte with their PPM (B) and PVM (C) still attached to the erythrocyte as indicated by the arrows. (D) Representative images of erythrocytes infected with ring stages of the NF54 RhopH2/Exp2 line treated with MβCD. Ring-stage parasites do not extrude out from erythrocytes. (E) Quantification of parasites remaining inside/outside erythrocytes after MβCD treatment was carried out by examining at least 100 different images of individual parasites for each experimental condition (100 for ring stages and 330 for trophozoites). (F, Top) Treatment of NF54 RhopH2/Exp2 trophozoites with 0.5 μM Cyt-D for 45 min prior to MβCD treatment does not inhibit parasite extrusion. (F, Bottom) Treatment with 0.5 μM Cyt-D alone does not cause extrusion of trophozoites but did alter PVM morphology (as indicated by arrows). (G, Left) Treatment with MβCD causes extrusion of late-stage P. falciparum- without causing erythrocyte rupture, unlike the control group where the trophozoite is still inside the erythrocyte (scale bar, 2 μM; direct magnification, ×5,000). (G, Right) Zoomed-in image of parasite extruded out of erythrocyte after MβCD treatment shows the PVM ruptured at multiple places indicated by black arrows. Electron microscopy of extruded P. falciparum. (G, Left) Lower-magnification views of control and MβCD-treated trophozoites. A higher-magnification view of an extruded parasite (right) shows that the PVM is compromised at multiple places (arrows) (scale bar, 500 nM; direct magnification, ×12,000).

A previous study showed that treatment of trophozoite-stage parasites with MβCD releases the parasites from the erythrocyte. In this study, the authors used filipin and ethidium bromide staining of glutaraldehyde-fixed parasites and did not assess the disposition of parasitophorous vacuolar membrane (PVM) and PPM in extruded parasites (13). We aimed to examine this effect in real time using time-lapse video microscopy, as well as to assess the disposition of PVM and PPM. For this purpose, we used two different transgenic P. falciparum lines; in one line, the gene encoding PPM-localized PfVP1 was tagged with mNeonGreen at its endogenous locus (kindly provided by Hangjun Ke [14]). The second line expressed two different fluorescently tagged proteins generated by using the CRISPR/Cas9 approach as described in Fig. 1A and B. The gene encoding PVM-localized EXP2 was tagged with mRuby at its endogenous location, and the gene encoding the RhopH complex protein RhopH2 was tagged with mNeonGreen (Fig. 1A and B). As shown in Fig. 1C, tagging these proteins with fluorescent markers did not affect their proper expression and localization. EXP2 was expressed in all stages and localized to the PVM, whereas RhopH2 was expressed at the highest level in mature stages of the parasite and localized to the punctate rhoptries in the schizont stage. RhopH2 is initially stored in merozoite rhoptries and secreted into the parasitophorous vacuole (PV) during invasion, eventually trafficking to the host cell membrane (15), as seen in ring stages (Fig. 1C). The use of these parasite lines permitted us to monitor the PPM, PVM, and rhoptries of the parasite when exposed to MβCD using live fluorescence microscopy. The trophozoites and early schizont stage-infected erythrocytes were attached to poly-l-lysine-coated plates and stained with fluorescent probes for the red blood cell (RBC) membrane and nuclei followed by treatment with MβCD for 30 min. MβCD was removed by washing with the culture medium, and the parasites were observed by live time-lapse fluorescent video microscopy. As shown in Fig. 2A and Video S1 in the supplemental material, the parasite extruded out of the host erythrocyte in a dramatic fashion. This expulsion occurred 10 to 15 min after MβCD was washed off, but the transition from being intracellular to being extracellular occurred in less than 10 s. The extruded parasites were surrounded by PPM (Fig. 2B, green) as well as the PVM (Fig. 2C; Fig. S1, red). Depending upon the plane from which the parasite emerges from the erythrocyte, parts of the PVM were observed still tethered to the host cell. In contrast to the late-stage parasites, cholesterol depletion of ring-stage parasites did not result in extrusion; RhopH2 was partially localized to the erythrocyte plasma membrane in the ring-stage parasites, but the parasite remained inside the host cell (Fig. 2D; Video S3). Quantification of this phenomenon showed that about 70% of the late-stage parasites extruded upon MβCD treatment (Fig. 2E).

The exclusion phenomenon was also observed by phase-contrast microscopy of the infected erythrocytes treated with 5 mM MβCD, which also showed that the late stages extrude out of the erythrocyte without causing lysis of the erythrocytes (Fig. S2). We assessed the integrity of the erythrocyte membrane by staining MβCD-treated cultures with fluorescent phalloidin. Phalloidin stains F-actin underlying the erythrocyte membrane when able to enter the cell. Parasite cultures not exposed to MβCD showed minimal phalloidin staining of either infected or uninfected erythrocytes. On the other hand, 60 to 70% of both uninfected and infected erythrocyte cytoskeletons were stained with phalloidin in MβCD-treated culture (Fig. S3). These observations suggest that MβCD causes a breach in the erythrocyte membrane in a manner that allows phalloidin to enter the cell but without causing catastrophic lysis that would release most of its cytosolic content. This breach in erythrocyte membrane was seen in ring stage-infected cells as well, yet this did not result in their expulsion.

The forceful expulsion of the parasite from the host cell remains mechanistically unexplained. MβCD treatment of mammalian cells has been shown to affect actin polymerization (16). Thus, one possibility could be that rearrangement and polymerization of RBC cytoskeletal actin may underlie the parasite extrusion. To test this, we treated trophozoite-infected erythrocytes with the actin polymerization inhibitor cytochalasin D (CytD) (17). Treatment with CytD prior to MβCD treatment did not inhibit MβCD-mediated extrusion of the parasites. Treatment with CytD alone did not cause the parasites to extrude out; however, we did observe defects in the PVM of the CytD-treated parasites (Fig. 2F; Video S2). Although CytD did affect the morphology of the PVM, it failed to prevent extrusion of the parasites when exposed to MβCD.

To assess the disposition of membranes of extruded parasites in some detail, we performed transmission electron microscopy of mature-stage parasites treated with MβCD. The extruded parasites were still surrounded by PVM, but the PVM was compromised at multiple places (Fig. 2G). The PPM, on the other hand, remained intact, with minimal changes in the intracellular structures. Images shown here are representative of multiple-electron micrographs.

### Prior treatment with novel antimalarials abrogates MβCD-mediated parasite extrusion.

**FIG 3:**
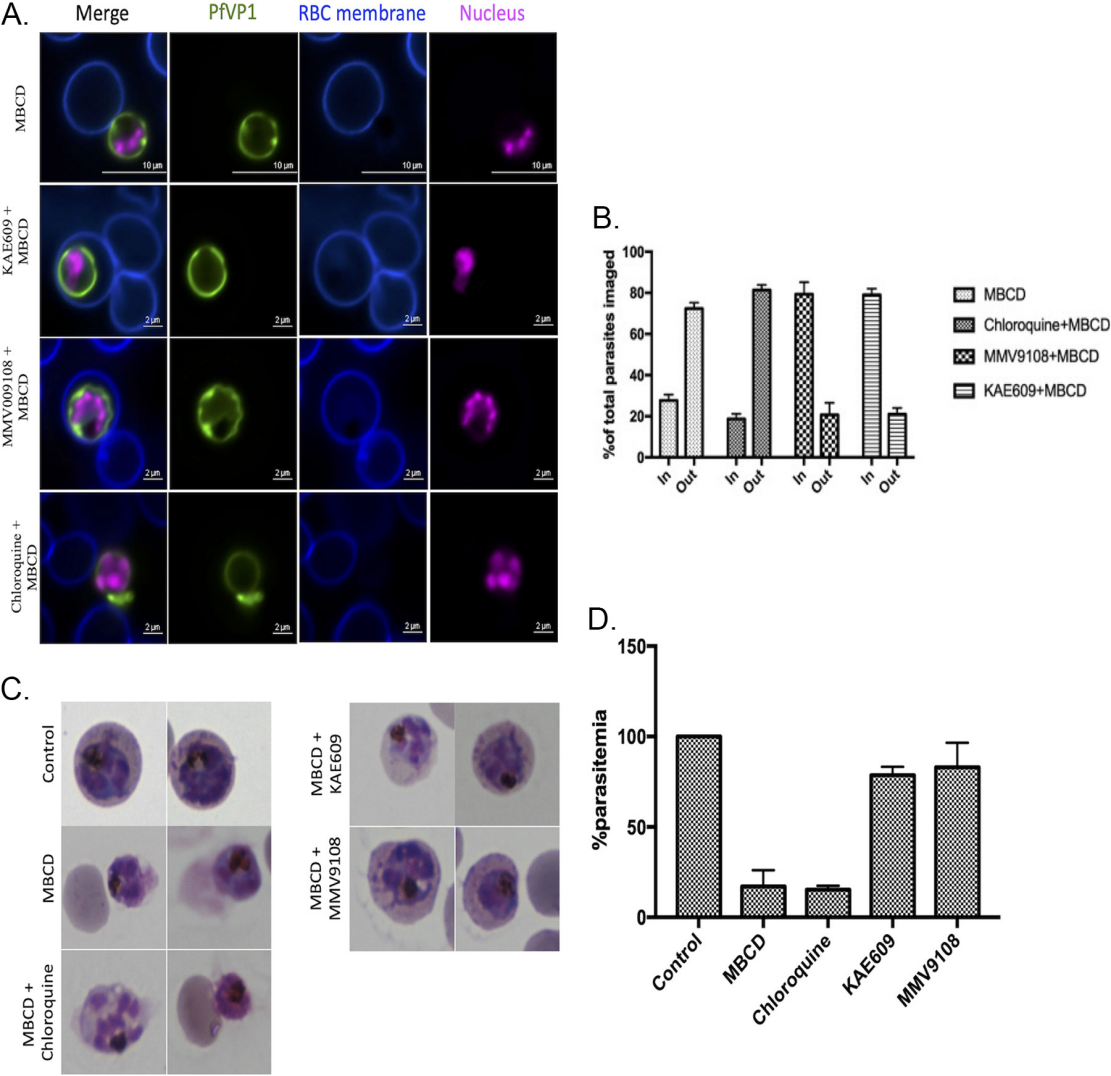
Treatment with PfATP4 or PfNCR1 inhibitors prior to MβCD treatment inhibited MβCD-mediated extrusion of parasites. (A) Trophozoite-stage parasites from the PfVP1-mNG line were treated with KAE609 (10 nM), MMV009108 (1 μM), and chloroquine (150 nM) prior to treatment with MβCD. Live fluorescent imaging showed that parasite extrusion was inhibited by prior treatment with KAE609 and MMV009108 (indicated by yellow arrows), while treatment with chloroquine like MβCD did not inhibit this extrusion (indicated by white arrows). Nucleus is stained with SYTO deep red (pink). Erythrocyte membrane is stained with WGA-Alexa 350 (blue). Representative images from 3 independent experiments for each condition. (B) Quantification from live fluorescence microscopy experiments of parasite extrusion upon drug treatment followed by MβCD treatment. Approximately 250 cells for each experimental condition over 4 independent experiment were assessed. (C) Giemsa-stained thin blood smears prepared from cultures after treatment with KAE609 (10 nM), MMV009108 (1 μM), and chloroquine (150 nM) prior to treatment with MβCD. (D) Quantitation of parasite extrusion upon drug treatment followed by MβCD treatment from Giemsa-stained thin blood smears (∼1,000 cells were counted).

We have previously shown that inhibition of PPM transporters, PfATP4 or PfNCR1, alters cholesterol dynamics in the PPM of late-stage parasites (9, 10, 18). We therefore examined the effects of these cholesterol homeostasis disruptors on MβCD-mediated extrusion phenomenon. We treated late-stage parasites for 2 h with KAE609 (a PfATP4 inhibitor), MMV009108 (a PfNCR1 inhibitor), or chloroquine (a heme detoxification inhibitor) followed by cholesterol depletion with MβCD. Interestingly, the parasite extrusion was greatly reduced by prior exposure to PfATP4 and PfNCR1 inhibitors but not by chloroquine (Fig. 3A). Quantitation of this phenomenon showed that 80% of parasites treated with dimethyl sulfoxide (DMSO) or chloroquine extruded following MβCD treatment, whereas only about 25% did so following treatment with PfATP4 and PfNCR1 inhibitors (Fig. 3B). This quantitation was also supported by observation of intracellular and extracellular parasites in Giemsa-stained thin blood smear (Fig. 3C and D). These results suggest that disruption of normal cholesterol dynamics in mature-stage parasites results in inhibition of MβCD-mediated extrusion of parasites.

### Role of erythrocyte plasma membrane cholesterol in invasion and the intraerythrocytic development of P. falciparum.

**FIG 4:**
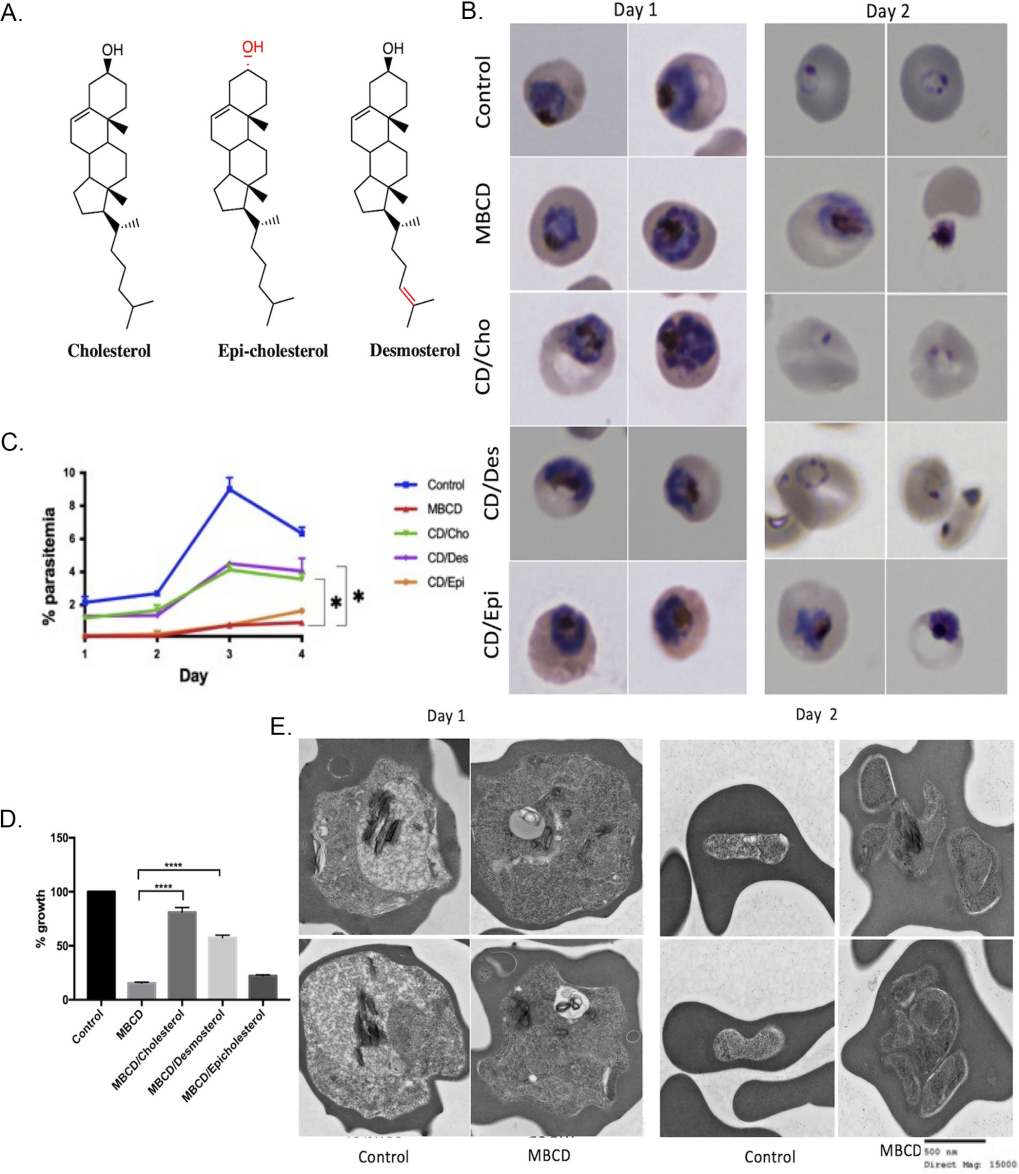
Cholesterol in the erythrocyte membrane is essential for growth and proliferation of P. falciparum. (A) Structures of MβCD, cholesterol, epicholesterol, and desmosterol. The differences between the structure of sterols are highlighted in red. (B) Giemsa-stained thin blood smears prepared from control, MβCD, and erythrocytes reconstituted with cholesterol, desmosterol, or epicholesterol at 24 and 48 h after treatment. (C) Parasitemia in 1:10 split of ring-stage parasites from control, MβCD (CD), or erythrocytes reconstituted with cholesterol (CD/Cho), desmosterol(CD/Des), or epicholesterol (CD/Epi) over 4 days (∼3,000 cells were counted). Based on one-tailed unpaired *t* test (*, *P* < 0.05). (D) Relative [^3^H]-hypoxanthine incorporation by ring-stage parasites over a 24-h period following treatment with MβCD or reconstituted with cholesterol, desmosterol, and epicholesterol. Based on unpaired *t* test with Welch’s correction (*, *P* < 0.05; ***, *P* < 0.001). Error bars, mean ± SD; *n* = 8. (E) Transmission electron microscopy (TEM) of the ring-stage erythrocyte treated with 5 mM MβCD and imaged after 24 or 48 h. (E, Left) After 24 h, in erythrocytes treated with MβCD, parasites are unable to form normal trophozoites. The nuclear membrane is indistinct, hemozoin is dispersed (indicated by arrows), and food vacuole is small compared to control erythrocytes. (E, Right) After 48 h, parasites in the control culture were able to invade the erythrocyte and form rings, while the parasites in the MβCD-treated erythrocytes did not progress further and appear to disintegrate (scale bar, 500 nM; direct magnification, ×15,000).

Previous studies have shown that reduction of erythrocyte plasma membrane cholesterol by treatment with MβCD resulted in inhibition of parasite growth (12). Since we found that the ring-stage parasites were not extruded from the erythrocyte by MβCD treatment, we went on to examine the effects of MβCD treatment of ring-stage parasites for their intraerythrocytic developmental cycle (IDC). In addition, we also examined the consequence of complementing cholesterol-depleted ring-stage parasites with different sterols such as cholesterol, desmosterol, or epicholesterol (structures shown in Fig. 4A). We treated the ring stage-infected erythrocytes with MβCD for 30 min followed by washes with normal culture medium. The development of ring-stage parasites was assessed by examination of Giemsa-stained thin smears. As shown in Fig. 4B, parasites in the control cells proceeded normally through the IDC and formed trophozoites and rings. In contrast, ring-stage parasites growing in cholesterol-depleted erythrocytes (MβCD treated) progressed to form what appeared to be trophozoites but failed to mature. Reconstitution with MβCD saturated with cholesterol (CD/Cho) or desmosterol (CD/Des) appeared to restore normal IDC progression. On the other hand, reconstitution with epicholesterol (CD/Epi) did not restore normal IDC, suggesting the importance of stereospecificity of cholesterol polar moiety in supporting parasite development. We also attempted to reconstitute with lanosterol or β-sitosterol but could not assess it since these resulted in lysis of erythrocytes. To assess the viability of the ring-stage parasites growing in MβCD-treated or sterol-reconstituted erythrocytes, we split the culture 1:10 into untreated normal erythrocytes. This was followed by measuring parasitemia over a subsequent 4-day period. As shown in Fig. 4C, ring-stage parasites growing in cholesterol-depleted or epicholesterol-reconstituted cells failed to propagate over the next two cycles. On the other hand, cholesterol- or desmosterol-reconstituted ring-stage parasites were able to propagate at a level of about 50% compared to the control ring-stage parasites. To assess progression of ring-stage parasites, we measured hypoxanthine incorporation over a 24-h period. As shown in Fig. 4D, there was a significant reduction in hypoxanthine incorporation in MβCD-treated and epicholesterol-reconstituted parasites, whereas ring-stage parasites in cholesterol- or desmosterol-reconstituted cells were able to incorporate hypoxanthine.

We examined the parasites by transmission electron microscopy at 24 and 48 h in control and following MβCD treatment. As expected at 24 h, the trophozoites in the control group displayed normal morphology with distinctive internal structures and normal hemozoin formation and went on to form ring stages at 48 h (Fig. 4E, top; Fig. S1). In contrast, MβCD-treated ring stages showed abnormal morphology with diminished food vacuoles at 24 h. These parasites failed to develop into normal schizonts and did not egress at 48 h after MβCD treatment (Fig. 4E, bottom; Fig. S4). These results suggest a defect in normal trophozoite development in erythrocytes with depleted cholesterol content.

**FIG 5:**
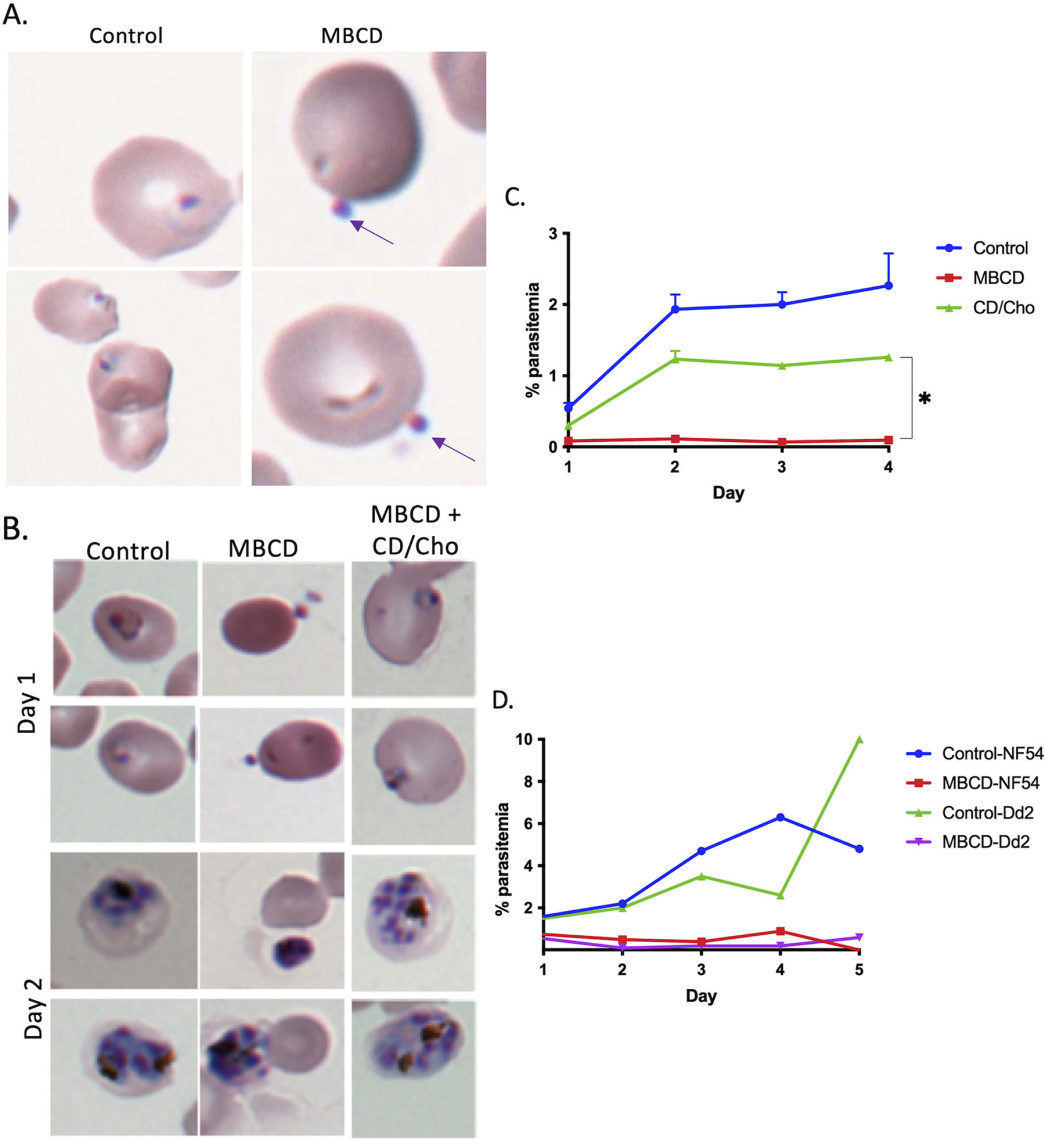
Reducing the cholesterol content in the erythrocyte membrane inhibits P. falciparum invasion. (A) Giemsa-stained thin blood smears from control and MβCD-treated erythrocytes after 24 h. While ring stages were seen in the control erythrocytes, no invasion was seen in the MβCD-treated erythrocytes, but merozoites appeared to remain attached to the surface as indicated by arrows. (B) Percoll-enriched trophozoites were added to erythrocytes treated with MβCD or reconstituted with cholesterol, and Giemsa-stained thin blood smears were prepared at 24 and 48 h. Reconstituted erythrocytes were able to support parasite growth. (C) Parasitemia from 1:10 split of culture from MβCD, treated and reconstituted with cholesterol in normal erythrocytes over a period of 4 days (∼3,000 cells were counted). Based on Mann-Whitney test. (*, *P* < 0.05). (D) Percoll-synchronized trophozoites from NF54 and Dd2 strains were added onto MβCD-treated erythrocytes. Parasitemia from 1:10 split of these cultures by examining Giemsa-stained thin blood smears (∼1,000 cells were counted).

We also assessed the ability of merozoites to invade and establish infection in MβCD-treated erythrocytes. MβCD-treated erythrocytes were added to Percoll-enriched schizonts. As shown in Fig. 5A, reduced cholesterol content in uninfected erythrocytes prevented merozoite invasion. The merozoites appeared to remain attached to the surface of MβCD-treated erythrocytes but failed to penetrate. Importantly, reconstitution of MβCD-treated RBCs with cholesterol complemented this invasion defect (Fig. 5B). Furthermore, the parasites were able to grow in these reconstituted erythrocytes and undergo normal IDC progression. Splitting the cultures 1:10 with addition of normal erythrocytes resulted in continued normal IDC progression of parasites from control and cholesterol-reconstituted cells but not from cholesterol-deficient cells (Fig. 5C).

Different P. falciparum strains invade using different pathways, a sialic acid-dependent or a sialic acid-independent pathway (19, 20). To see if cholesterol in the erythrocyte membrane is selective for a given pathway, we added schizonts of either NF54 (sialic acid-dependent) or Dd2 (sialic acid-independent) strains to MβCD-treated erythrocytes. Merozoites from neither strain were able to invade MβCD-treated erythrocytes (Fig. 5D).

## DISCUSSION

Using live video microscopy of fluorescently tagged parasites, we have extended previous observations showing expulsion of trophozoite-stage P. falciparum upon treatment with MβCD. We also showed that this expulsion is inhibited by new antimalarial compounds that disrupt cholesterol homeostasis in the parasite. The dramatic expulsion of the trophozoite-stage parasites following MβCD treatment remains unexplained. It is hard to perceive how an intracellular parasite can escape out of the host erythrocyte without causing catastrophic lysis of the host membrane in response to reduction in the cholesterol content in the erythrocyte plasma membrane. Expulsion of the parasite occurs after 40 to 45 min exposure to MβCD and after MβCD is washed off. Actual expulsion, however, occurs within seconds. The force with which the expulsion appears to occur suggests an active process mediated either by the host or the parasite. Our observation that cytochalasin D did not prevent the expulsion of trophozoites indicates that actin polymerization is not required for this process. Osmotic pressure buildup through solute uptake in trophozoites resulting in their release from the host cells has been widely used for synchronization of blood-stage P. falciparum. However, this requires incubation with a high (about 0.5 M) concentration of solutes such as sorbitol. At 5 mM concentration, MβCD exposure does not pose an osmotic challenge, and at ∼1,300-Da molecular weight, MβCD would not be taken up by the trophozoite-infected erythrocyte through its new permeability pathway. Furthermore, sorbitol-mediated expulsion of the parasite occurs within a few minutes and is accompanied by catastrophic lysis of the infected erythrocyte. In contrast, expulsion caused by MβCD-mediated cholesterol depletion occurs after 40 to 45 min when MβCD is no longer present, and the infected erythrocyte does not undergo lysis. Also, the MβCD-mediated expulsion is inhibited by prior treatment with PfATP4 and PfNCR1 inhibitors. These observations seem to argue against an osmotic imbalance as the force underlying the expulsion phenomenon described here. We speculate the possibility of a hitherto unrecognized process through which the parasite might sense cholesterol content of the erythrocyte membrane. It is of interest to note that MβCD-mediated expulsion of the parasite resembles expulsion of fungal pathogens from the mammalian cells through a process termed vomocytosis wherein the expulsion does not result in lysis of the host cell (21–23).

Inhibition of the parasite expulsion by compounds that inhibit PfATP4 or PfNCR1 was unexpected; this inhibition was not a result of parasite demise since chloroquine failed to do so. We have previously shown that both these inhibitors induce cholesterol accumulation in the PPM in a reversible manner (10). PfATP4 inhibition results in Na^+^ influx into the parasite and a collapse of Na^+^ gradient across the PPM. We propose that PfNCR1 requires a Na^+^ gradient to maintain cholesterol homeostasis, and its collapse by PfATP4 inhibition results in indirect inhibition of PfNCR1. The net result of both PfATP4 and PfNCR1 inhibition, therefore, is a disruption of cholesterol homeostasis in late-stage P. falciparum. We envision a dynamic movement of cholesterol between various membranes of infected erythrocytes. Recent investigations have also supported the notion of cholesterol gradients in infected erythrocytes (24–28). Reduction of the plasma membrane cholesterol content thus appears to be sensed by the parasite in a manner that results in the expulsion of trophozoites.

The erythrocyte plasma membrane contains large amounts of cholesterol (50 mol% of total lipids). Upon infection by *Plasmodium*, the PVM acquires cholesterol, but the PPM remains highly deficient in cholesterol. Indeed, this fact is the basis for widely used saponin-mediated freeing of intraerythrocytic parasites. Previous studies have suggested the importance of cholesterol for P. falciparum growth and invasion (12, 13, 25). A recent study using lattice light-scattering microscopy demonstrated the importance of erythrocyte membrane remodeling for the formation of the PVM, a process in which cholesterol is likely to play a significant role (11). We observed that ring-stage parasites growing in cholesterol-deficient erythrocytes fail to fully progress. Furthermore, the digestive vacuoles of trophozoite-like parasites at 24 h after MβCD treatment of ring stages had unusual morphology (Fig. 3E and Fig. S1 in the supplemental material). At 48 h after MβCD treatment of ring stages, parasites appeared to attempt segmentation without being able to form mature merozoites (Fig. 3E and Fig. S1). One possible explanation could be that cholesterol dynamics assist lipid and/or fatty acid import to sustain parasite growth. The fact that cholesterol and desmosterol (but not epicholesterol) could substantially rescue these defects suggests stereospecificity in the role of cholesterol to sustain parasite growth. Further studies will be required to gain mechanistic insights into cholesterol dynamics and their contribution to parasite growth.

## MATERIALS AND METHODS

### Parasite lines and culture conditions.

Experiments were carried out using the following different P. falciparum-tagged lines; (i) the 3D7 strain in which endogenous PfVP1 gene was tagged with mNeonGreen (mNG) at the C-terminal end (PfVP1-mNG) (14), used for monitoring the dynamics of the parasite plasma membrane; and (ii) the NF54 strain in which endogenous RhopH2 gene was tagged with mNG and the endogenous Exp2 gene tagged with mRuby3 (NF54 Rhoph2/Exp2). Cloning was carried out with Infusion (Clontech) and NEBuilder HiFi (NEB). To generate an endogenous EXP2-mRuby3 fusion, 5′ and 3′ homology flanks targeting the 3′ end of *exp2* were PCR amplified from plasmid pyPM2GT-EXP2-mNG (29) using primers CACTATAGAACTCGAGGGAGAAACAATCTTTTATATAAAATGTACAGAGTTTGAAAG and TCCTCCACTTCCCCTAGGTTCTTTATTTTCATCTTTTTTTTCATTTTTAAATAAATCTCCAC and inserted between XhoI and AvrII sites in the plasmid pbPM2GT (30). The mRuby3 coding sequence was then amplified from plasmid pLN-HSP101-SP-mRuby3 (31) using primers GATGAAAATAAAGAACCTAGGGGAAGTGGAGGAGTG and TAACTCGACGCGGCCGTCACTTGTACAGCTCGTCCATGCC and inserted between XhoI and AvrII, resulting in the plasmid pbEXP2-mRuby3. This plasmid was linearized at the AflII site between the 3′ and 5′ homology flanks and cotransfected with pUF-Cas9-EXP2-CT-gRNA into NF54^attB^::HSP101^DDD^ (31). Parasites were maintained in 10 μM trimethoprim (TMP) to stabilize the HSP101^DDD^ fusion, and selection with 2.5 μg/mL blasticidin S was applied 24 h posttransfection. A clonal line bearing the EXP2-mRuby3 fusion was derived by limiting dilution after the parasite returned from selection and designated NF54^attB^::HSP101^DDD^+EXP2-mRuby3.

For generation of an endogenous RhopH2-mNG fusion, a genomic RNA (gRNA) target site was chosen upstream of the *rhoph2* stop codon (TCTTCACTGATTTCTTTGTA), and the gRNA seed sequence was synthesized as a sense and antisense primer pair (sense shown) TAAGTATATAATATTTCTTCACTGATTTCTTTGTAGTTTTAGAGCTAGAA, annealed, and inserted into the AflII site of the plasmid pAIO3 (32), resulting in the plasmid pAIO3-RhopH2-CT-gRNA. To integrate mNG at the 3′ end of *rhoph2*, a 5′ homology flank (up to, but not including, the stop codon) was amplified from NF54^attB^ genomic DNA using primers AATTTCATCATTATGAAAGTTCTCAGCTTAAGAAGCATATATTAAGAATATAGTTTCAGA and CCTCCACTTCCCCTAGGACTGCTCTTCAGAATATACAGGTTTTTTATAAGATCCTCCGATATCTCCTTATATGGATCAGATATATCTGAGAAA, incorporating several synonymous mutations in the seed sequence of the gRNA target site within the *rhoph2* coding sequence. A 3′ homology flank (beginning at the endogenous stop codon) was amplified using primers GTGACACTATAGAACTCGAGTAAACGTTAAAAAAAAAATATATATAAGGAGAAAGCACTG and TCTGAAACTATATTCTTAATATATGCTTCTTAAGCTGAGAACTTTCATAATGATGAAATT, assembled in a second PCR using primers GTGACACTATAGAACTCGAGTAAACGTTAAAAAAAAAATATATATAAGGAGAAAGCACTG and CCTCCACTTCCCCTAGGACTGCTCTTCAGAATATACAGGTTTTTTATAAGATCCTCCGATATCTCCTTATATGGATCAGATATATCTGAGAAA, and inserted between XhoI and AvrII sites in pyPM2GT-EXP2-mNG (29), resulting in the plasmid pyPM2GT-RhopH2-mNG. This plasmid was linearized at the AflII site between the 3′ and 5′ homology flanks and cotransfected with pAIO3-RhopH2-CT-gRNA into NF54^attB^::HSP101^DDD^+EXP2-mRuby3. Selection with 2 μM DSM-1 was applied 24 h posttransfection (along with 10 μM trimethoprim). After parasites returned from selection, a clonal line containing the EXP2-mRuby3 and RhopH2-mNG fusions was obtained by limiting dilution and designated NF54^attB^::HSP101^DDD^+EXP2-mRuby3+RhopH2-mNG. Correct integration of the transgenes was confirmed by PCR amplifications using the following primers (indicated in Fig. 1): A, CCAGAATGTTTCGGACCATGTAC; B, TGGTATCCGGAGCCATCTACCATG; C, CACTTTGTAACTTCATTTTCTAAAATGACCTTGTTC; D, GCAACAAGTGCCTTAACCACCG; E, CGATGACTTTGATCCTCATGGTTTGC; and F, TCACTTATGTTGTATAGAGACACAATTCGT.

This line was used for monitoring the parasitophorous vacuolar membrane and to mark the parasite. P. falciparum parasites were cultured in O-positive (O^+^) human blood from Interstate Blood Bank, Tennessee, in RPMI 1640 supplemented with 2 g/L sodium bicarbonate, 10 mg/L hypoxanthine, 15 mM HEPES, 50 mg/L gentamicin sulfate, and 0.5% AlbuMax II. Parasite culture was maintained at 2.5% hematocrit at 37°C in 90% N_2_, 5% CO_2_, and 5% O_2_.

### Preparation of MβCD and MβCD-sterol complexes.

Parasites were treated with 5 mM MβCD diluted in appropriate medium from a stock solution of 25 mM MβCD (catalog no. 377110050; Thermo Fisher Scientific) in phosphate-buffered saline (PBS) in appropriate medium. Sterols (cholesterol (Sigma; catalog no. C3045-25G), desmosterol (Steraloids; catalog no. C3150-000), and epicholesterol (Steraloids; catalog no. C6730-000]) were loaded onto MβCD according to previously published protocol (33). Briefly, 10 μL of 15-mg/mL sterols dissolved in ethanol was added to 500 μL 5% (wt/vol) MβCD, heated for 10 min at 80°C, and mixed by inverting several times until the solution was clear. The above step was repeated 4 times to add a total of 50 μL sterol stock solution. The solution was heated until the sterols were stably incorporated in MβCD (clear solution). MβC-sterol complexes were snap frozen in dry ice for 2 min. Subsequently, the MβCD-sterol complexes were lyophilized in a speed vac until all liquid had evaporated and a fluffy powder remained at the bottom of the tube and stored at −20°C. Immediately before use, 375 μL of medium was added to the MβCD-sterol complexes and vortexed until dissolved. The MβCD-sterol complexes were sterilized using a 0.22-μm syringe filter.

### Treatment of parasitized erythrocytes with MBCD and reconstitution with MBCD-sterol complexes.

Parasite cultures were synchronized by treatment with 0.5 M alanine for 10 min. Erythrocyte infected with ring-stage parasites were treated with 5 mM MβCD for 30 min at 37°C and washed 3 times with prewarmed RPMI 1640 media. Hematocrit was maintained at 2.5% throughout the culture and washing steps. The parasites were returned to normal culture conditions. In experiments involving reconstitution with MβCD-sterol complexes, parasites were first treated with MβCD as described above. Following this, the cells were incubated with a 1:7 dilution of MβCD-sterol complexes for 30 min. Parasites were washed 3 times and returned to normal culture conditions. Giemsa-stained thin blood smears were prepared 24 and 48 h after the treatment with MβCD or after reconstitution with MβCD-sterol complexes. The culture at 48 h was split 1:10 in normal erythrocytes. Growth of the parasites in these cultures was followed for 4 days by examining Giemsa-stained thin blood smears.

### Hypoxanthine incorporation of ring-stage erythrocytes treated with MβCD or MβCD-sterol complexes.

Ring-stage erythrocytes (1.5% parasitemia) were treated with MβCD for 30 min and washed 3 times with normal medium. The hematocrit was maintained at 2.5%. MβCD-treated ring-stage erythrocytes were then treated with either MβCD-cholesterol, MβCD-desmosterol, or MβCD-epicholesterol for 30 min and washed 3 times with low-hypoxanthine medium. The hematocrit was then adjusted to 1.5% by addition of low-hypoxanthine medium to the cells. We then plated 200 μL of cells in triplicates in a 96-well plate. Each well was pulsed with 22 μL of 0.5 μCi/mL [^3^H]-hypoxanthine (PerkinElmer; product no. NET177) and incubated for 24 h at 37°C in a 5% CO_2_, 5% O_2_, and 90% N_2_ chamber. Parasites were lysed by freeze-thaw and were collected on filters using a cell harvester (PerkinElmer Life Sciences). This was followed by addition of MicroScint-O scintillation cocktail, and incorporation of [^3^H]-hypoxanthine was measured using a TopCount scintillation counter (PerkinElmer Life Sciences).

### Parasite invasion in MβCD- or MβCD-cholesterol-treated erythrocytes.

We treated 500 μL of 50% hematocrit erythrocytes with 5 mM MβCD for 30 min followed by 3 washes. In addition, similarly treated erythrocytes were reconstituted with MβCD-cholesterol for 30 min followed by 3 washes. Synchronized late-stage trophozoites were enriched by centrifugation over a 70% Percoll cushion (3,000 rpm, 20 min) and washed thrice with medium. Enriched late-stage trophozoites were added to MβCD-treated, MβCD-cholesterol-reconstituted, and control erythrocytes. Giemsa-stained thin blood smears were prepared 24 and 48 h following the addition. The culture at 48 h was split 1:10 in normal erythrocytes. Growth of the parasites in these cultures was followed for 4 days by examining Giemsa-stained thin blood smears.

### Live microscopy of parasites.

Glass bottom culture dishes (35 mm) were coated with 0.1% poly-l-lysine overnight and washed 3 times with PBS. Parasite culture (250 μL at 2.5% hematocrit) was added to the dishes and incubated for 30 min to allow attachment. Culture dishes were washed 3 times with PBS followed by addition of medium. For MβCD extrusion experiments with the NF54 Rhoph2/Exp2 line, all washes, incubation, and imaging were done in medium supplemented with TMP. Parasites were attached to poly-l-lysine-coated plates and incubated in RPMI containing wheat germ agglutinin-Alexa 350 (WGA-350) (Invitrogen; catalog no. W7024) at 5 μg/mL final concentration and incubated for 12 min to stain the erythrocyte plasma membrane. In addition to WGA staining, the PfVP1-mNG line was also stained with SYTO deep red nuclear stain for 30 min. Parasites were treated with 5 mM MβCD followed by three washes with medium. The medium was replaced with phenol red-free medium followed by live fluorescence microscopy. Imaging was done using the Nikon Ti microscope. WGA-Alexa 350 was visualized using a DAPI (4′,6-diamidino-2-phenylindole) filter set, mNeonGreen with (fluorescein isothiocyanate) FITC, and SYTO deep red nuclear stain with Cy5, and mRuby was visualized using tetramethyl rhodamine isocyanate (TRITC) filter set. Video microscopy parameters are given in the legends of movies in the supplemental material.

### Inhibition of MβCD-mediated extrusion by novel antimalarials.

To look at the effects of antimalarials on MβCD-mediated extrusion, parasites were stained with WGA-350 and SYTO deep red for the PfVP1-mNG line or WGA-350 for the NF54 Rhoph2/Exp2 line as described above and treated with 10× 50% effective concentrations (EC_50_s) of chloroquine (150 nM), KAE609 (10 nM), or MMV009108 (1 μM) for 2.5 h prior to 30 min treatment with MβCD and washed thrice with medium. Medium supplemented with drugs (at the concentration mentioned above) was added back to the dish, and live fluorescent imaging was carried out. Quantification of parasites remaining inside/outside erythrocytes was carried out by examining at least 100 different images of individual parasites for each experimental condition.

### Cyt-D treatment prior to MβCD treatment of parasites.

Attached NF54 RhopH2/Exp2 trophozoites were stained with WGA-350 for 12 min, followed by three washes. The cells were then treated with 0.5 μM Cyt-D for 45 min. This was followed by the addition of 5 mM MβCD for 30 min. The culture dishes were washed 3 times, and live fluorescence microscopy was performed to examine parasite extrusion. In the control condition, parasites were treated with Cyt-D without MβCD treatment.

### Transmission electron microscopy of trophozoite-infected, MβCD-treated erythrocytes.

Erythrocytes infected with trophozoite-stage parasites were treated with 5 mM MβCD for 30 min at 2.5% hematocrit followed by three washes with PBS and incubated in complete medium for 30 min at 37°C. Cells were washed once in PBS by gentle centrifugation and fixed with 2% paraformaldehyde and 2.5% glutaraldehyde in 100 mM cacodylate buffer and were fixed for 1 h at room temperature. Cells were gently pelleted by centrifugation, resuspended in cacodylate buffer, and immediately stored on dry ice for shipment.

Ring stage-infected erythrocytes were treated with 5 mM MβCD for 30 min, washed thrice with PBS, and returned to culture at 37°C. At 24 and 48 h after the treatment, infected erythrocytes were fixed and prepared for transmission electron microscopy as described above.

### Phalloidin staining of MβCD-treated cultures.

Infected erythrocyte cultures from the PfVP1-mNG line were treated with 5 mM MβCD for 30 min at 37°C and washed 3 times with prewarmed RPMI media. Phalloidin staining was carried out as described (34). Briefly, the MβCD-treated cultures were stained with phalloidin-Alexa 594 (Invitrogen; diluted 1:50 from a 200-U mL^−1^ stock in methanol) for 5 min at 37°C and washed thrice with prewarmed phenol red-free RPMI 1640. The cells were added to the glass slides and sealed with greased coverslips. Images were taken using the Nikon Ti microscope. mNeonGreen was visualized using the FITC channel, and Phalloidin Alexa 594 was visualized using the TRITC filter set.
