# Supplementary material for: Dramatic Consequences of Reducing Erythrocyte Membrane Cholesterol on Plasmodium falciparum
Source: Microbiol Spectr. 2022 Feb 23;10(1):e00158-22. doi: 10.1128/spectrum.00158-22 (PMC8865471; doi:10.1128/spectrum.00158-22)
Supplement: SUPPLEMENTAL FILE 4 — Supplemental material. Download SPECTRUM00158-22_Supp_4_seq6.pdf, PDF file, 3.7 MB [file spectrum00158-22_supp_4_seq6.pdf]

Supplementary Material

Dramatic consequences of reducing erythrocyte membrane cholesterol on *Plasmodium falciparum*

Avantika I. Ahiya[a], Suyash Bhatnagar[a], Joanne Morrisey[a], Josh R. Beck[b], Akhil B. Vaidya[a*]

a. *Center for Molecular Parasitology, Institute for Molecular Medicine and Infectious Disease, Department of Microbiology and Immunology, Drexel University College of Medicine, Philadelphia, Pennsylvania 19129*

b. *Department of Biomedical Sciences, Iowa State University, Ames, Iowa 50011*

*To whom correspondence should be addressed at Center for Molecular Parasitology, Dept. of Microbiology and Immunology, Drexel University College of Medicine, 2900 Queen Lane, Philadelphia, PA 19129. Tel.: 215-991- 8557; Fax: 215-848-2271;

E-mail: av27@drexel.edu

Supplementary Videos Legends

Supplementary video 1: Treatment with MßCD causes extrusion of trophozoites from erythrocytes

Representative time lapse video microscopy of the NF54 RhopH2/Exp2 line after MßCD treatment. Erythrocyte membrane is stained with WGA-Alexa-350 (Blue). Infected erythrocytes were subjected to live fluorescent microscopy after treatment with MßCD. Parasites extrude out from erythrocyte without causing lysis of the erythrocyte. Video was acquired using the Nikon Ti microscope. The interval between each frame was 4 sec and the video was captured for a total 20 min (93 frames, FPS:0.09, frames 59-64 shown in the video). Images were deconvoluted by Richardson Lucy algorithm using the Nikon NIS elements software package and converted to .avi files.

Supplementary video 2: Cyt-D treatment does not inhibit MßCD mediated parasite extrusion

Representative time lapse video microscopy of the NF54 RhopH2/Exp2 line after MßCD treatment. Erythrocyte membrane is stained with WGA-Alexa-350 (Blue). Infected erythrocytes were subjected to live fluorescent microscopy after treatment with Cyt-D followed by treatment with MßCD. Treatment with Cyt-D does not inhibit MßCD mediated extrusion of the parasite. Video microscopy was performed as mentioned above for video 1. The interval between each frame was 31 sec and the video was captured for a total 5 min (11 frames, FPS:0.04, all 11 frames shown in the video)

Supplementary video 3: Treatment with MßCD does not cause extrusion of ring stage parasitesErythrocytes infected with ring stages of the NF54 RhopH2/Exp2 were treated with

MßCD . Erythrocyte membrane is stained with WGA-Alexa-350 (Blue). Ring stage parasites do not extrude out after MßCD treatment. Video microscopy was performed as mentioned for video 1. The interval between each frame was 31 secs and the video was captured for a total 5 mins (11 frames, FPS:0.04, all 11 frames shown in the video)

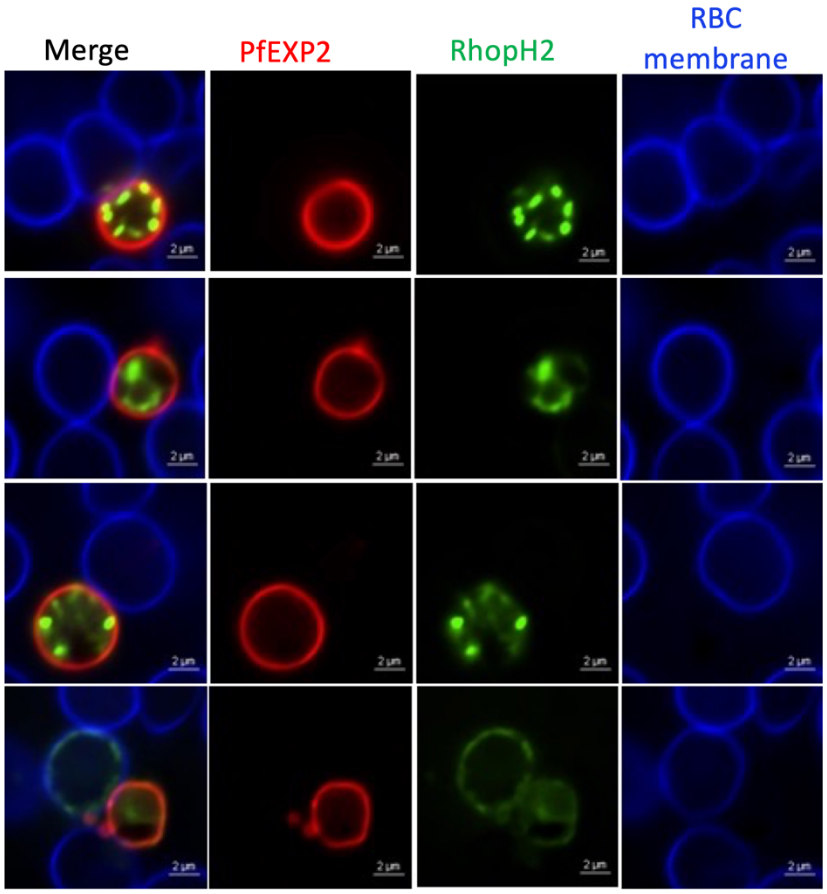

Supplementary Figure 1: Images of extruded parasites following MßCD treatment of of the NF54 RhopH2/Exp2 transgenic line.

Representative images (out of >50) of live NF54 RhopH2/Exp2 line after MßCD treatment. Erythrocyte membrane is stained with WGA-Alexa-350 (Blue), EXP2 is red and RhoH2 is green. Infected erythrocytes were subjected to live fluorescent microscopy after treatment with MßCD. Scale bar is 2 µm.

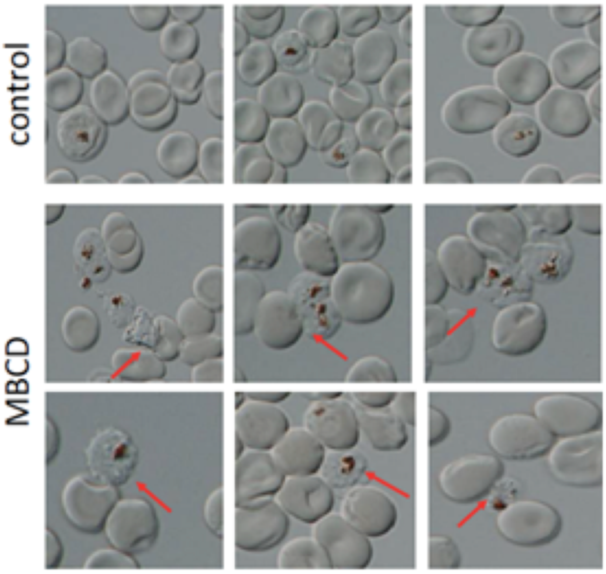

Supplementary figure 2: Phase contrast images of late stage *P. falciparum* extruded after treatment with MßCD.

Infected erythrocytes were subjected to phase contrast microscopy after treatment with MßCD. Extruded parasites are indicated by red arrows.

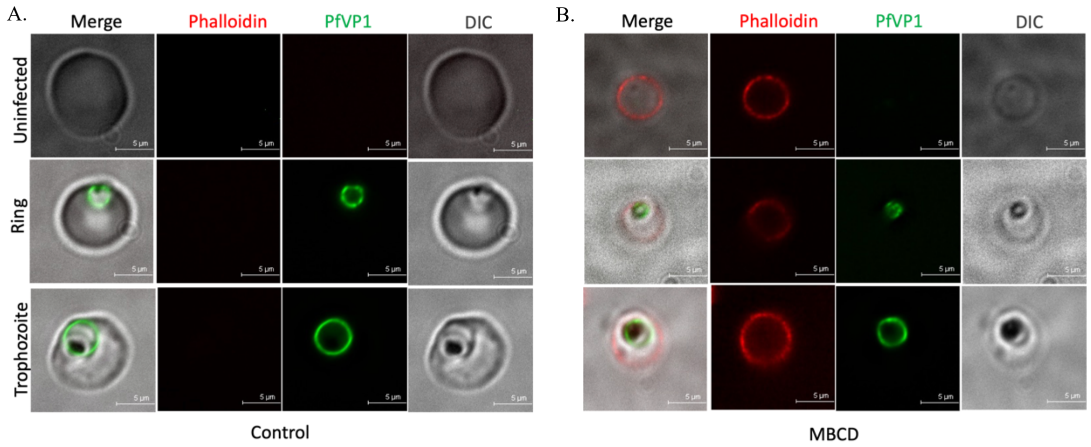

Supplementary figure 3: Phalloidin staining of MßCD treated cultures from PfVP1-NG transgenic line: (A) Representative images of uninfected and infected cells from untreated cultures after staining with Phalloidin AlexaFluor 594. (B) Representative images of uninfected and infected cells from MßCD treated cultures followed by staining with Phalloidin AlexaFluor 594. (Scale bar- 5 µm). Overall, 60-70% of uninfected as well as infected erythrocyte cytoskeletons were stained with phalloidin, indicating membrane breach without the lysis of erythrocytes.

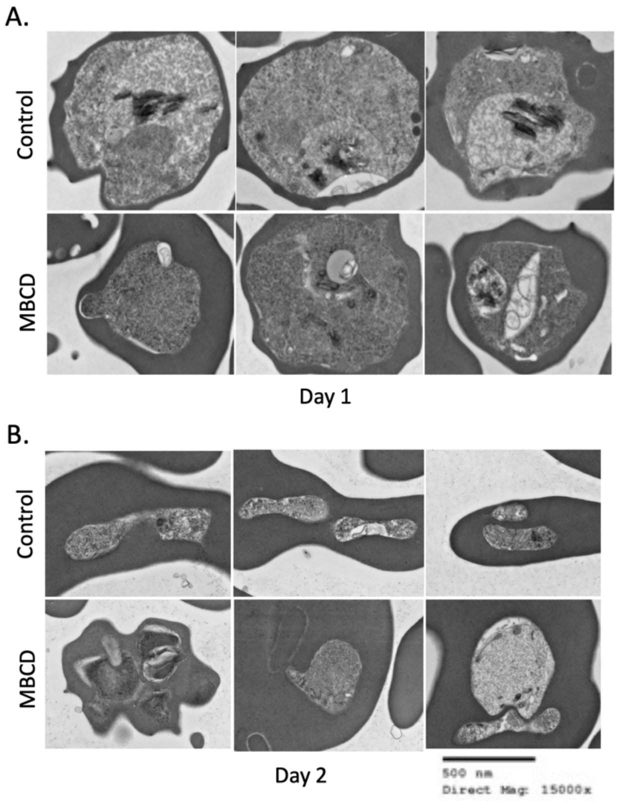

Supplementary figure 4: Transmission electron microscopy of the ring stage erythrocyte treated with 5mM MßCD and imaged after 24 (Day 1) or 48 hours (Day2)
(A) After 24 hours, the trophozoite like parasites in the MßCD treated cultures (lower panel) have indistinct membranes, compromised food vacuole and dispersed hemozoin compared to the controls (Upper panel) (B) After 48 hours, in the control culture (Upper panel) the parasites invaded and formed rings while the parasites in the MßCD treated cultures (Lower panel) failed to form mature merozoites (Scale bar-500nM, Direct magnification-15,000x).
